# The Effects of *MMP3* (rs679620) and *VDR* (rs731236) Gene Polymorphisms on Dental Caries: A Pilot Study

**DOI:** 10.1055/s-0044-1787978

**Published:** 2024-07-19

**Authors:** Seda Özmen, Pınar Yılmaz Atalı, Ömer Birkan Ağralı, Beste Tacal Aslan, Özlem Özge Yılmaz, Tolga Polat, Korkut Ulucan

**Affiliations:** 1 Department of Restorative Dentistry, Yeditepe University Faculty of Dentistry, Istanbul, Turkey; 2 Department of Restorative Dentistry, Marmara University Faculty of Dentistry, Istanbul, Turkey; 3 Department of Periodontology, Marmara University Faculty of Dentistry, Istanbul, Turkey; 4 Department of Basic Medical Sciences, Marmara University Faculty of Dentistry, Istanbul, Turkey

**Keywords:** dental caries, gene polymorphism, MMP3, VDR

## Abstract

**Objective** Caries formation is a process affected by various factors. Studies have shown that genetic factors also play a role in caries formation. The aim of our study is to examine the effects of matrix metalloproteinase ( *MMP)3* (rs679620) and vitamin D receptor ( *VDR* ) (rs731236) gene polymorphisms on caries formation.

**Materials and Methods** Following routine oral examinations in individuals aged between 20 and 44 years, the diagnosis was made according to the decayed, missing, and filled teeth (DMFT) index, and experimental group was defined as “high caries risk” (DMFT ≥ 14, *n* = 28), and the control group as “no caries” (DMFT = 0, *n* = 28). Plaque index and bleeding on probing were measured from participants with a detailed anamnesis. Periodontally healthy individuals with less than 10% bleeding on probing were included in the study ( *n* = 56). After DNA isolation from blood samples taken from the participants, the genotyping of *MMP3* (rs679620) and *VDR* (rs731236) gene polymorphisms were determined using the real-time polymerase chain reaction technique.

**Statistical Analysis** Data were analyzed with IBM SPSS V23.0. Data distribution was evaluated with Kolmogorov–Smirnov's test. Pearson's chi-square test was used to compare categorical data according to groups. The results were evaluated using a significance level of *p* < 0.05.

**Results** Regarding *MMP3* and *VDR* gene polymorphisms, there was a statistically significant difference between the groups in terms of *MMP3* (rs679620) ( *p* < 0.001). There was no statistically significant difference between the *VDR* (rs731236) genotype distributions of the groups ( *p* = 0.659).

**Conclusion** Within the limits of this study, *MMP3* rs679620 gene polymorphism may have an effect on caries formation.

## Introduction

Dental caries is one of the most common diseases observed worldwide, occurring as a result of the interaction of biofilm, diet, time, and host. 1 2 Despite current knowledge of the various risk factors for dental caries susceptibility, there might be individual variations that can help explain why some people exposed to the same risk factors develop disease, while others do not. Therefore, the difference in caries susceptibility in the general population suggests that immunological and genetic factors play an important role in its pathogenesis. 1 2 3 All this information shows that dental caries is a multifactorial disease. As a result of the genomic scan performed in 2008 aiming to detect the caries-associated genomic regions, gene regions associated with low and high caries susceptibility were identified. 4

Single nucleotide polymorphisms (SNPs), the modern unit of genetic variation, are defined as changes occurring in a single base pair in the DNA sequence. SNPs used for genetic studies are observed in every 200 to 300 base pairs throughout the genome. 5 6 SNPs provide important benefits to indicate the susceptibility of individuals to the disease, differences in response to treatment, and clinical dimensions of diseases. 7 Many studies have been conducted to investigate the genetic predispositions of dental caries lesion formations using SNPs. As a result of the studies performed so far, it is thought that SNPs in tooth mineralization, immune system, salivary protein, and taste receptor genes may affect dental caries. Researchers have conducted many studies on SNPs in genes encoding proteins that play an important role in enamel formation. 8

In recent studies, host-derived matrix metalloproteinases (MMPs) have been detected in the oral cavity. 9 As a result, it has been determined that several MMPs regulate the mineralization stage by controlling the proteoglycan transformation during tooth development. 10 The enamel matrix also contains other important matrix components, such as proteinases. Many enamel proteins are degraded by proteinases shortly after secreted into the enamel. The proteinases responsible for these early cleavage events regulate enamel biomineralization by converting enamel proteins into cleavage products. 11

Vitamin D is a regulator of mineral homeostasis mediated by calcium absorption, which can affect the quality of bone, enamel, and dentin. It has quite an important role for the regulation of the mechanisms of phosphate and calcium ions, each with their parts in the protection and strengthening of teeth. 6 12 1,25(OH)2D3, the most biologically active metabolite of vitamin D, acts by binding to an intracellular vitamin D receptor (VDR). The gene encoding VDR, located on chromosome 12q13.11, contains several polymorphic regions. Several studies have evaluated the possible association between this *VDR* gene polymorphisms and increased susceptibility to periodontitis. In some studies, the allele and genotype frequencies of the *VDR* *Taq* I polymorphism were analyzed in patients with dental caries in Turkish and Chinese populations. 6 13 14

The aim of this pilot study is to examine the effects of polymorphisms in the *VDR* (rs731236) and *MMP3* (rs679620) genes that play a role in tooth mineralization, and on the formation of caries in individuals of different caries risk groups.

## Materials and Methods

### Selection of the Subjects

Inclusion criteria for the study were:

- Being between 20 and 44 years old.
- Not having any genetic inherited disease in herself/himself or her/his first degree relatives
- Having no systemic disease and no regular drug use.
- Plaque index ≤1.
- Bleeding on probing index ≤ 10%.

Exclusion criteria for the study were:

- Having received radiotherapy/chemotherapy treatment.
- Undergoing hormone therapy.
- Recent vitamin D supplement use.
- Using any medication that affects salivary flow rate and buffering capacity.
- Undergoing orthodontic treatment.
- Having any genetic inherited disease in herself/himself or her/his first degree relatives.

The decayed, missing, and filled teeth (DMFT) index was used to determine the previous caries experience, with the clinical and radiological evaluations according to the World Health Organization (WHO) criteria. Teeth with caries lesions in any part of the tooth were scored as (D), the teeth that were extracted due to caries were scored as (M), while the restored teeth without secondary caries, and those restored with crowns or bridge abutments due to caries were scored as (F). Aside from teeth with caries lesions, all wisdom teeth and those extracted due to esthetic concerns, trauma, and periodontal disease were not included in the calculations. Obtained DMFT values were recorded in subject registration forms.

### Calculation of Plaque Index and Bleeding on Probing

All subjects who participated in the study were examined by a faculty member dentist and a research assistant dentist to standardize the classification.

The “plaque index scoring,” developed by Silness and Löe, 15 was used to determine the amount of supragingival microbial dental plaque. After the teeth were isolated with cotton pads and air dried, microbial dental plaque near the gingival margin on four surfaces was examined both visually and with an examination probe. Bleeding on probing measurements were performed (six sites per tooth; PCP-12 probe), and periodontally healthy individuals with less than 10% of bleeding areas were included in the study.

**Fig. 1:**
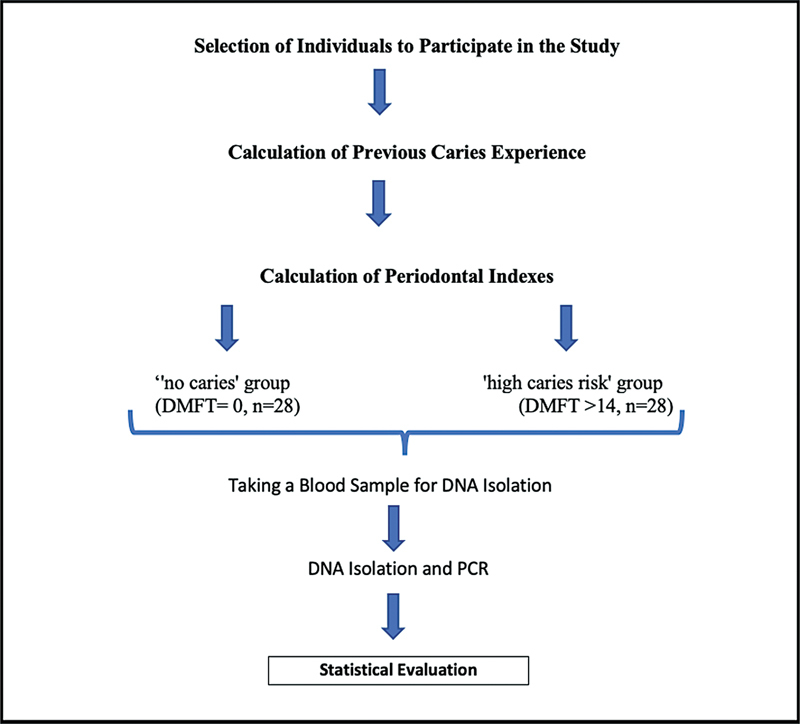
Study protocol. DMFT, decayed, missing, and filled teeth; PCR, polymerase chain reaction.

A total of 236 individuals aged between 20 and 44 years, who applied to a state university's dental school's hospital between the approval date of the ethics committee (February 3, 2023) and (March 20, 2023), were diagnosed according to DMFT index, following routine oral examinations. As a result of the examination, 56 people who met the study criteria were included in the study and then were divided into two groups according to the presence of caries lesions ( Fig. 1 ).

The experimental group thus consisted of 28 subjects in the “high caries risk” group (DMFT ≥ 14), and the control group consisted of 28 subjects (DMFT = 0) “no caries lesions” ( *n* = 56).

### Collecting Blood Samples for Genetic Analysis

Venous blood samples of study subjects were drawn from the most suitable antecubital vein and transferred into the EDTA tubes at the dental school and hospital of a state university blood transfusion center. Following blood drawing, the tubes were turned upside down 8 to 10 times, as recommended by the manufacturer, to ensure complete mixing of the additive and the blood. The drawn blood was stored in the refrigerator at 4°C for genetic analysis.

### Genetic Analysis

DNA isolation was performed with the G-spin total DNA isolation kit (Intron Biotechnology, South Korea) using 200 μL of peripheral blood drawn from the subjects. Genotyping of the *VDR* rs731236 (GeneBank accession number NM_000376.3, base change: c.1056T > C, amino acid change: p.Ile352 = ) and *MMP3* rs679620 (GeneBank accession number NM_002422.5, base change: c.1385C > G, amino acid change: p.Ala462Gly) polymorphisms were performed using real-time polymerase chain reaction (RT-PCR) on a StepOnePlus (Thermo Fisher Scientific, Inc., United States) device and Taqman SNP Genotyping Assays genotyping kits according to the manufacturers' protocols (cat. no. 4351379, Thermo Fisher Scientific, Inc.).

**Table 1:** Sequences of the TaqMan probe used for genotyping of the *VDR* rs731236 and *MMP3* rs679620 polymorphism

		Sequence, 5′-3′
***VDR*** **rs731236**	VIC/FAM	TGGACAGGCGGTCCTGGATGGCCTC **[A/G]** ATCAGCGCG GCGTCCTGCACCCCAG
***MMP3*** **rs679620**	VIC/FAM	CTAACAAACTGTTTCACATCTTTTT **[C/T]** GAGGTCGTA GTAGTTTTCTAGATAT

Note: The TaqMan probe sequence used in the detection of SNPs in the studied gene region, the base change region in the polymorphism is shown in bold.

A and G alleles were determined using VIC and FAM primers for *VDR* rs731236, respectively. C (G) and T (A) alleles were determined using VIC and FAM primers for *MMP3* rs679620, respectively ( Table 1 ). For a total volume of 10 µL reaction, 5 µL of Genotyping Master Mix (Applied Biosystems, Foster City, California, United States), 3.5 µL of nuclease-free H _2_ O (Thermo Fisher Scientific, Inc.), 0.5 µL of genotyping test (Applied Biosystems), and 1 µL of DNA (20 ng) were used.

### Statistical Evaluation

Data were analyzed with IBM SPSS V23.0 (IBM Corp. Released 2012. IBM SPSS Statistics for Windows, Armonk, New York, United States: IBM Corp.). Mann–Whitney's *U* -test was applied for age, plaque index, and bleeding on probing variables. Data distribution was evaluated with Kolmogorov–Smirnov's test. Pearson's chi-square test was used to compare categorical data according to groups in gene polymorphism data. Statistical analysis of univariate logistic regression results of *MMP3* rs679620 and *VDR* rs731236 polymorphisms on caries risk was performed with IBM SPSS 25.0 program (IBM SPSS Statistics for Windows: IBM Corp.). The significance level was taken as *p* < 0.050.

## Results

No statistically significant difference was observed when age, plaque index, and bleeding at probing index data were compared between the groups ( Table 2 ).

**Table 2:** Differences between groups across scores of environmental factors

	Variables		
	Age	Plaque index	Bleeding on probing
**No caries group (** ***n*** ** = 28)**	25.28 ± 3.25	0.08 ± 0.16	0.02 ± 0.15
**High-risk group (** ***n*** ** = 28)**	26.64 ± 7.13	0.04 ± 0.07	0.07 ± 0.26
***p*** - **Value**	0.699	0.892	0.529

Note: Mann–Whitney's *U* -test, *p* < 0.05.

When *MMP3* and *VDR* gene polymorphisms were compared between the groups, a statistically significant difference was found between the groups regarding *MMP3* distributions ( *p* < 0.001). The GA ratio was 21.4% in “no caries group” and 75% in “high-risk group.” The GG ratio was 50% in “no caries group” and 10.7% in “high-risk group.” While the dominant G allele was found in 60.7% of the no caries group and 48.2% of the high-risk group, the recessive A allele was found to be 39.3% in the no caries group and 51.8% in the high-risk group. GG genotype and G allele were found to be higher in the no caries group, and GA genotype and A allele were found to be higher in the high-risk group. No statistically significant difference was observed in terms of allele frequency between the no caries group and the high-risk group ( *p* = 0.1814) ( Table 3 ). As seen in Table 4 , when examining the univariate logistic regression results of *MMP3* rs679620 polymorphism on caries risk, it was determined that having the GA genotype increases the risk of caries by 16.33 times (odds ratio [OR]: 16.33, 95% confidence interval [CI]: 3.49–76.35), and having the GA + AA genotype increases the risk by 8.33 times (OR: 8.33, 95% CI: 2.04–34.17).

**Table 3:** Genotype distribution and allelic frequency of the polymorphism of *MMP3* rs679620 among no caries group and high-risk group

	**Genotype**			***p*** **-Value**	**Allelic frequency**		***p*** **-Value**
	**GG**	**GA**	**AA**	**<0.001**	**G**	**A**	0.181
**No caries group (** ***n*** ** = 28)**	14	6	8		34	22	
**Percentage**	50.0%	21.4%	28.6%		60.7%	39.3%	
**High-risk group (** ***n*** ** = 28)**	3	21	4		27	29	
**Percentage**	10.7%	75.0%	14.3%		48.2%	51.8%	

Notes: Pearson's chi-square test; significance was evaluated as at least *p* < 0.05. Comparison of control group was made by using the chi-square test. Statistically significant difference between the groups is emphasised in the bold part.

**Table 4:** Univariate logistic regression results of *MMP3* rs679620 polymorphism on caries risk

	OR (95% CI)	*p* -Value
GG (ref.)		**0.001**
GA	16.33 (3.49–76.35)	**<0.001**
AA	2,33 (0.41–13.17)	0.337
GA + AA vs. (GG ref.)	8.33 (2.04–34.07)	**0.003**
AA vs. (GG + GA ref.)	0.42 (0.11–1.59)	0.200

Abbreviations: CI, confidence index; OR, odds ratio.

Note: *p* < 0.05 indicates the statistically significant difference. Statistically significant difference between the groups is emphasised in the bold part.

Regarding the *VDR* distributions. while the dominant A allele was found in 66.1% of the no caries group and 33.9% of the high-risk group, the recessive G allele was found to be 57.1% in the no caries group and 42.9% in the high-risk group. Statistically no significant difference was observed between the no caries group and the high-risk group in terms of genotype ( *p* = 0.659) and allelic frequency ( *p* = 0.331) ( Table 5 ). As seen in Table 6 , the univariate logistic regression results of VDR rs731236 polymorphism on caries risk were not found to be statistically significant ( *p* > 0.05).

**Table 5:** Genotype distribution and allelic frequency of the polymorphism of *VDR* rs731236 among the no caries group and high-risk group

	**Genotype**			***p*** **-Value**	**Allelic frequency**		***p*** **-Value**
	**AA**	**AG**	**GG**	0.659	**A**	**G**	**0.331**
**No caries group (** ***n*** ** = 28)**	13	11	4		37	19	
**Percentage**	46.4%	39.3%	14.3%		66.1%	33.9%	
**High-risk group (** ***n*** ** = 28)**	10	12	6		32	24	
**Percentage**	35.7%	42.9%	21.4%		57.1%	42.9%	

Notes: Pearson's chi-square test; significance was evaluated as at least *p* < 0.05. Comparison of control group was made by using the chi-square test. Bold text is used to emphasise the statistical result.

**Table 6:** Univariate logistic regression results of *VDR* rs731236 polymorphism on caries risk

	OR (95% CI)	*p* -Value
AA (ref.)		0.661
AG	1.42 (0.44–4.53)	0.555
GG	1.95 (0.43–8.83)	0.386
AG + GG vs. (AA ref.)	1.56 (0.53–4.56)	0.416
GG vs. (AA + AG ref.)	1.64 (0.41–6.58)	0.488

Abbreviations: CI, confidence index; OR, odds ratio.

Note: *p* < 0.05 indicates the statistically significant difference.

## Discussion

Dental caries is one of the most common chronic diseases among children and adults. The direct cost of treatment is estimated to account for an average of 4.6% of global health expenditures. 16

It is commonly understood that dental caries, a prevalent illness in developing nations, has a higher occurrence among specific demographic groups. Dental caries is a frequently encountered disease in epidemiology that requires medical intervention. Vipeholm study suggests that an individual's resistance to cavities is associated with a high rate of cariogenic nutrients in one's diet. It is believed that susceptibility or resistance to caries may be due to the combination of genotypic, phenotypic, and environmental influence.

The aim of our study was to investigate the effects of *MMP3* (rs679620) and *VDR* (rs731236) gene polymorphisms, which may be among the genotypic factors, on caries formation. For this purpose, we wanted to standardize the environmental factors between the groups and observe the effect of the genetic factor. When the age, plaque index, and bleeding on probing data were compared between the groups, no statistically significant difference was observed. When we look at the results in terms of genetics, it was observed that *MMP3* rs679620 polymorphism was effective in caries formation; *VDR* rs731236 polymorphism had no effect.

This study has some limitations; first of all, it has a low sample size since it is only a pilot study. We tried to standardize the individuals in terms of only plaque index and bleeding on probing index; however, since carious lesion formation is a multifactorial disease, standardization of other factors would also provide more accurate results for future studies.

Existing studies have identified a large number of SNPs associated with caries, and only a few of these SNPs have been discussed in studies conducted in the Turkish population. 17 18

Many genes are involved in the development of tooth enamel. Vitamin D is a fat-soluble steroid necessary to maintain the mineral balance of the body. 19 It plays an important role in the calcification of enamel and dentin, susceptibility to dental caries, and gingivitis and in the immune response to mouth microbial infections. 20

The function and biological activity of vitamin D is modulated by its interaction with the VDR protein, and the activity of the VDR protein is affected by polymorphisms of the *VDR* gene. The *VDR* gene has been found to affect the activity of an important vitamin D metabolite involved in the formation of tooth enamel, suggesting its possible impact on the risk of dental caries. 20

Although results from individual studies have been inconsistent, a meta-analysis of controlled clinical trials showed that early vitamin D supplementation can reduce the risk of dental caries by 47 to 54%. 21

In the last decade, both pro and active forms of host-derived MMPs have been identified in the oral cavity. MMPs contribute to various physiological processes such as embryonic development, tissue turnover, and wound healing. In addition to these, they are also involved in pathological processes such as cancer, cardiovascular disease, arthritis, periodontitis, and fibrosis. 10 It has been suggested that several MMPs have a role in tooth development and may regulate mineralization by controlling the proteoglycan cycle. 22

It has been shown that genes involved in dentin formation, such as *MMP2* and *MMP3* , may contribute to faster carious lesion progression in dentin and periapical pathologies. 10

The transcriptional level of most *MMPs* is regulated by growth factors and cytokines, but it has also been shown that the SNPs of several *MMP* genes are transcriptional regulators. The two SNPs analyzed in a recent study had previously been associated with the susceptibility of the host to developing periapical lesions in individuals with untreated carious lesions. 10

In our pilot study, we investigated whether the polymorphisms observed in *MMP3* and *VDR* genes are associated with caries lesions in individuals with similar periodontal health. Understanding the genetics of susceptibility or resistance to caries lesion formation will provide a new perspective on the caries formation process and facilitate the development of strategies to prevent carious lesion formation.

According to the WHO age classification made in 2017, 18 to 65 years of age is defined as a young individual. Considering the time of completion of tooth development and enamel maturation stage and the changes observed in the mouth with age progression, we narrowed the age range and included individuals between the ages of 20 to 44 years in our study. 23

Radiotherapy and chemotherapy treatments, as well as systemic diseases and most of the medications used to treat these conditions may cause changes in the salivary flow rate. Therefore, patients with systemic disease, patients on regular drug use, and those receiving radiotherapy treatment were excluded from the study. Individuals with orthodontic appliances were also excluded from the study due to the fact that the use of the appliance may cause changes in the plaque ratio and the saliva pH levels. 24 25

Mimicking the previous genetic studies to determine the effect of genetics on caries formation, we included the individuals with a DMFT value of zero as the no caries group, and the individuals with a DMFT value of more than 14 as the high-caries risk group.

DNA isolation is the first step in the application of molecular diagnostic methods. Thus, in our study, blood samples were used for the isolation of genomic DNA due to the lower risk of contamination and the ease of obtaining a more concentrated DNA. 17

RT-PCR systems are more advantageous over other techniques since the nonspecific amplifications do not affect the analysis results, real-time analyses can be performed by these systems, with fast cycle times and shortened processing times in addition to high sensitivity, specificity, and reproducibility. 26 Therefore, we also preferred to use the RT-PCR device in our study.

In the studies performed to evaluate the effects of genetics on dental caries formation, various gene polymorphisms were studied that are thought to be associated with the quality and quantity of the saliva, taste-bud receptors, tooth mineralization, and the immune system. 4

While these studies focused on genes in a single group (e.g., only mineralization-related genes or immunity-related genes), studies investigating gene polymorphisms from different groups were also conducted. It was stated that future research should be done to support the findings obtained in these studies. 18 27 28 Therefore, we aimed to investigate the association of *VDR* rs731236 and *MMP3* rs679620 gene polymorphisms, which are in the mineralization-related group, with caries.

There was a statistically significant difference in between the *MMP3* genotype distributions of the study groups ( *p* < 0.001).

When studies on *MMP3* polymorphisms in the past 20 years were examined, the authors could isolate only two studies that evaluated the rs679620 region. In a study conducted by Karayasheva et al in 2016 with Bulgarian students, no significant relationship was found in between the *MMP3* rs679620 gene polymorphism and caries formation. 10 In a more recent study by Borilova Linhartova et al in 2020 on children in the Czech population, no correlation was similarly found between *MMP3* rs679620 polymorphism and caries formation, whereas a significant relationship was found between *MMP20* rs1711437 polymorphism and caries formation. 29 In these studies, periodontal standardization (plaque index and bleeding at probing measurements were performed, periodontally healthy individuals were included in the study, and no significant difference was found between the groups in terms of these measurements) was not provided between the groups as in our study, and our study was performed on a different ethnic group (Turkish). The authors believe that the difference in our study could be associated with this fact. 10 29

There was no statistically significant difference in between our study groups regarding the *VDR* genotype distributions ( *p* = 0.934).

When the studies on *VDR* polymorphisms were analyzed, the authors were able to identify six studies evaluating the rs731236 region. Parallel to our findings, Kong et al (2017), Qin et al (2019), Izakovicova Holla et al (2017), and Yu et al (2017) have also stated that the *VDR* rs731236 gene polymorphism was not associated with caries formation. 13 14 30 31 In contrast, in the study conducted by Cogulu et al (2016), among children aged between 6 and 12years in Turkish society, and in the study by Hu et al (2015), conducted among 30- to 67-year-old adults in the Chinese population, a relationship between the *VDR* rs731236 gene polymorphism and the caries formation was suggested. We think that the difference in the study by Cogulu et al might be due to the sample made up of pediatric population unlike our sample group, though both studies were conducted in a Turkish ethnicities. Hu et al's study was performed on individuals with Chinese origin, and thus, this may also explain this difference. 2 17

In our study, one of the reasons for obtaining different results from studies examining the same gene polymorphism region is that only periodontally healthy individuals were included in the study.

Besides different polymorphism regions of *VDR* and *MMP3* , other genes associated with tooth mineralization could also be investigated to see if there exists a relationship with carious lesion formation for future research.

## Conclusion

Within the limits of this pilot study, a relationship was found in between the *MMP3* rs679620 polymorphism and caries formation, yet there was no relationship in between the *VDR* rs731236 polymorphism and caries formation.
